# A network analysis of anxiety and depression symptoms among empty nesters in China

**DOI:** 10.3389/fpsyg.2025.1667813

**Published:** 2025-09-18

**Authors:** Hong Luo, Tao Wang

**Affiliations:** The First Affiliated Hospital of Dalian Medical University, Dalian, Liaoning, China

**Keywords:** empty nester, anxiety, depression, network analysis, China

## Abstract

**Background:**

Mental health is closely linked to the development of various diseases and serves as a cornerstone of healthy aging. Empty nesters, who lack family support, are particularly vulnerable to mental health issues such as anxiety and depression. Network analysis offers a novel methodological approach to uncovering associations between mental disorders. This study aimed to construct a network model of anxiety and depression symptoms among Chinese empty nesters, identify central and bridge symptoms, and explore their interrelationships to inform targeted interventions.

**Methods:**

A total of 5,130 individuals aged 60 and above were selected from the China Longitudinal Healthy Longevity Survey (CLHLS 2017–2018). Depression and anxiety symptoms were assessed using the 10-item Center for Epidemiologic Studies Depression Scale (CESD-10) and the Generalized Anxiety Disorder Scale-7 (GAD-7). A symptom network was constructed using the Extended Bayesian Information Criterion (EBIC) model and the Graphical Gaussian Model (GGM) with the Least Absolute Shrinkage and Selection Operator (LASSO) regularization. Central symptoms and bridge symptoms were identified using Expected Influence (EI) and bridge Expected Influence (bEI). The stability and accuracy of the network were evaluated through non-parametric bootstrap methods. Additionally, the Network Comparison Test (NCT) was employed to examine potential gender differences in network structure.

**Results:**

Network analyses revealed that the central symptoms of anxiety-depression network were CESD-3 (I felt sadness), GAD-2 (Not being able to stop or control worrying) and GAD-4 (Trouble relaxing). CESD-1(I was bothered by things that do not usually bother me), GAD-1 (Feeling nervous, anxious, or on edge) and GAD-3 (Worrying too much about different things) are critical bridge symptoms linking depression and anxiety. Furthermore, this study found that the anxiety-depression network among empty nesters did not exhibit gender differences.

**Conclusion:**

This study identified CESD-3 (I felt sadness), GAD-2 (Not being able to stop or control worrying), and GAD-4 (Trouble relaxing) as central symptoms in the anxiety-depression network among empty nesters. These findings provide critical insights for developing precise interventions aimed at mitigating the progression of anxiety and depression, improving mental health in this population, and ultimately promoting healthy aging.

## 1 Introduction

Population aging has become a pressing global challenge, with China experiencing particularly rapid demographic shifts. The accelerated aging process, coupled with evolving family structures, has positioned China as the nation with the world’s largest elderly population (Man et al., 2021). Concurrently, a growing proportion of older adults now fall into the “empty nester” category (Yan et al., 2022) – those whose adult children have left home for education, employment, or permanent relocation. Research indicates that by 2030, the proportion of empty nester in China will reach 90%, with an estimated over 200 million elderly people becoming empty nesters (Zhang et al., 2019). Whether by choice or circumstance, these elderly individuals increasingly live independently while confronting multifaceted challenges, including deficiencies in daily care and emotional support (Xu and Yang, 2023). Chronic emotional loneliness, sustained social isolation, and persistent health concerns collectively create a vulnerable psychological state that predisposes empty nesters to anxiety, depression, and related mental health disorders, this consequently leads to adverse health outcomes, such as an increased incidence of falls, malnutrition, safety accidents, and poor medication adherence. These negative outcomes impair the quality of life for the elderly and hinder the achievement of active aging (Hou, 2025).

A substantial body of research has demonstrated that empty nesters are at a higher risk of developing anxiety and depressive symptoms. Meta-analysis findings indicate that the prevalence of depression among older adults in China is 31.17% (Wu et al., 2024), with empty nesters exhibiting an even higher rate of 43% (Song et al., 2023). This trend is further supported by longitudinal social surveys in China, which confirm that empty nesting constitutes a significant risk factor for depression in the elderly (Zheng et al., 2022). Anxiety symptoms among empty nesters often arise from declining physical and social functioning, as well as diminished communication abilities (Zheng and Zhang, 2024). Moreover, empty nesters experience heightened levels of loneliness, a well-established risk factor for anxiety, which explains their increased susceptibility to anxiety disorders (Ciuffreda et al., 2021). Although anxiety and depression are distinct conditions, they share considerable neurobiological overlap (Hettema, 2008). Liu et al. reported a comorbidity rate of 9.0% between the two disorders (Liu et al., 2022), and their co-occurrence leads to more severe health consequences—including reduced quality of life, elevated disability risk, and increased mortality—compared to either condition alone (Zhao et al., 2024).

Among empty nesters, anxiety and depression rarely exist in isolation. They are more like a “symbiotic pair,” intertwining and mutually reinforcing each other, forming a more complex and persistent state of comorbidity compared to other age groups (Sillero et al., 2023). While anxiety and depression in general adults may stem from work, financial, or marital pressures, the core driver for empty nester is “loss”—anxiety arises from the fear of “future loss,” while depression stems from mourning “past loss.” Moreover, their symptomatic presentation differs from that of younger adults: empty nester often exhibit somatization symptoms such as palpitations, chest tightness, headaches, and loss of appetite, which further impair their physical health (Divers et al., 2022; Zhang Y. et al., 2023). Additionally, elderly individuals with comorbid anxiety and depression demonstrate poorer cognitive performance, including memory decline, slowed reaction times, and difficulty concentrating, closely resembling dementia (Kryza-Lacombe et al., 2024). As an easily overlooked group, empty nesters face urgent challenges related to comorbid depression and anxiety disorders. A comprehensive understanding of the interplay between depression and anxiety in this population, along with the development of targeted intervention strategies, is crucial for mitigating adverse outcomes at the individual, familial, and societal levels.

To effectively address these challenges, it is necessary to investigate the specific symptom-level interactions between anxiety and depression. Grounded in network analysis theory, this study provides a conceptual framework aimed at enhancing our understanding of the nature and mechanisms of mental disorders by modeling and analyzing the complex system of symptom relationships (Borsboom, 2017). In this model, symptoms are represented as nodes, and their causal or associative relationships are represented as edges (Borsboom and Cramer, 2013). This approach allows for the identification of the most influential core symptoms and bridge symptoms that facilitate comorbidity within the network (Hofmann et al., 2016). These highly influential symptoms are critical for treating the disorders and determining intervention targets.

Although network analysis has been successfully applied to various populations, including students, healthcare workers, and patients with chronic diseases (Chen et al., 2023; Zheng et al., 2024; Yohannes et al., 2022), and these studies consistently indicate a strong association between anxiety and depression, while revealing significant differences in network structure according to age, gender, and environmental factors, different populations exhibit different network configurations at different times (Schlechter et al., 2021). However, current research on anxiety and depression among empty-nest elderly mainly relies on traditional methods (Soqia et al., 2022), primarily focusing on the broad categories of anxiety or depression, with little attention paid to the interrelationships of symptoms at the symptom level.

To address this gap, the present study utilizes network analysis to examine the relationships between depression and anxiety symptoms in Chinese empty nesters. By identifying central and bridge symptoms within the network, this research aims to enhance our understanding of the complex interplay between anxiety and depression in this population, thereby providing clinicians with evidence-based insights to develop targeted intervention strategies.

## 2 Methods

### 2.1 Study setting and participants

This study used data from the China Longitudinal Healthy Longevity Survey (CLHLS 2017–2018) (Peking University Center for Healthy Aging and Development Studies, 2020). CLHLS is a study of older adults in China conducted by the Centre for Healthy Ageing and Development Studies (CHADS) at Peking University in 1998. Through a multi-stage random sampling method, seven surveys were conducted between 2000 and 2018, covering 23 provinces across China, and primarily collecting information on participants’ daily lives, demographics, behaviors, and health-related characteristics. CLHLS was conducted by the Campus Institutional Review Board of Duke University (Pro00062871) and the Biomedical Ethics Committee of Peking University (IRB00001052-13,074) for approval and adoption, the study followed the Declaration of Helsinki, and informed consent was obtained from all participants.

**Figure 1:**
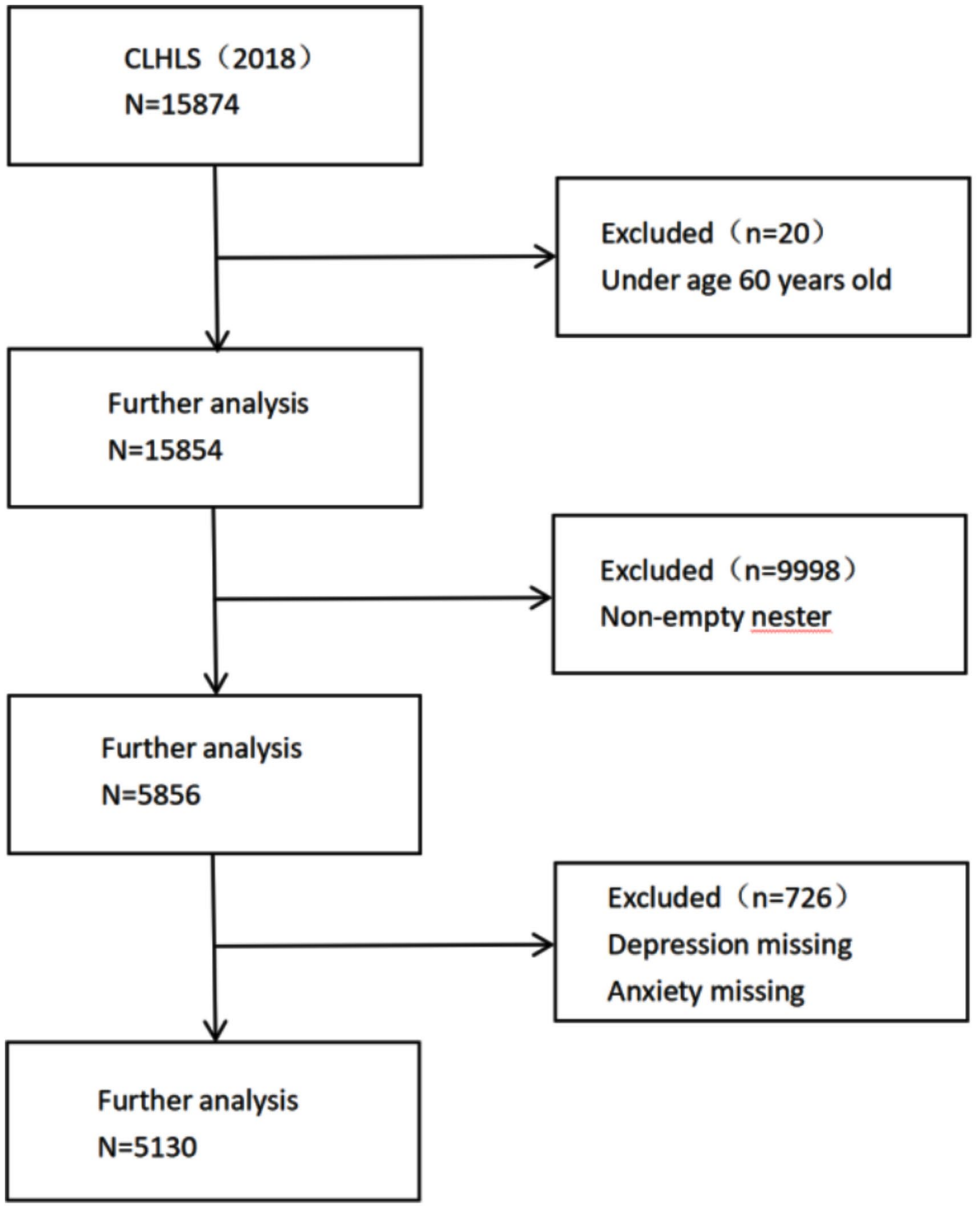
Flow diagram of the selection of sample.

Inclusion criteria for this study were (1) age ≥60 years, and (2) empty nester: living alone or with spouse only. Exclusion criteria: (1) missing data such as anxiety and depression scales. A total of 5,130 study participants were finally included for analysis. The screening process is shown in Figure 1.

### 2.2 Measurement

#### 2.2.1 Empty nester

Empty nester (Yu-Ting et al., 2022) defined as living alone or having children who have left the home to live with their spouse only, and in the questionnaire, these individuals self-reported their living situation as living alone or only with their spouse.

#### 2.2.2 Anxiety

In this study, we measured the severity of anxiety symptoms over a two-week period in older adults by means of the Generalized Anxiety Disorder scale-7 (GAD-7), which has been widely used among Chinese older adults (He et al., 2010). The scale consists of seven items, each scored between 0 and 4, where 0 means never, 1 means a few days, 2 means more than half of the days, and 3 means almost every day, and ultimately, scores on the scale range from 0–21, with higher scores being associated with more severe anxiety symptoms. A score greater than 5 is considered to indicate significant levels of anxiety symptoms (Spitzer et al., 2006).

#### 2.2.3 Depression

The CLHLS used the 10-item Center for Epidemiologic Studies Depression Scale (CESD-10) to assess depression in older adults (Chen and Mui, 2014). The scale consists of 10 items, and subjects were asked to assess the frequency of each outcome item during the previous week, with a score of 0 for seldom or never, 1 for sometimes, 2 for often, and 3 for always. Item 5, “Are you full of hope for future life, “item 7, “Do you feel as happy as you are when you are young “and item 10, “How is your sleep quality now” were all reverse-scored before any analysis to ensure consistency in the direction of all items. The higher the final total score, the more severe the depressive symptoms. The scale has been widely used among Chinese older adults, a score of 10 or higher indicates a state of depression (Boey, 1999).

### 2.3 Network estimation

#### 2.3.1 Network analysis

This study used R for data analysis. A network of depression and anxiety symptoms was constructed using the extended Bayesian information criterion (EBIC) model and the graphical Gaussian model (GGM) with the visual least absolute shrinkage and selection operator (LASSO) from the R packages “qgraph” and “bootnet.” Where EBIC is used to select the best fitting model, GGM is used to check the ordered or categorical data and LASSO is used for regularization (Epskamp et al., 2018). Each symptom in the network model is represented as a node, the line connecting two nodes is called an edge, and the color and thickness of the edge indicates the correlation between the two symptoms and its degree (Epskamp et al., 2012).

#### 2.3.2 Centrality and stability

Centrality index is often used to measure the core symptoms of a network model. In this study, there is a negative correlation between the nodes, so we used Expected influence (EI) to quantify the central symptom, the higher the EI value of a node, the higher its influence (Epskamp et al., 2012). In addition, we used the bridge function in the R package “Network Tools” to calculate the bridge expected influence (bEI). bEI is used to quantify the bridge symptom between two communities, the higher the bEI value, the more crucial role this symptom plays in connecting with other symptoms (Jones et al., 2021). The extent to which a node’s variance can be explained by all its neighboring nodes is referred to as its predictability. In this study we calculated the predictability of each symptom through the R program “mgm,” which is represented by a pie chart of the ring around the node, where highly predictable nodes are more susceptible to the influence of nearby nodes (Haslbeck and Waldorp, 2015). The stability and accuracy of the network model was assessed by the R package “bootnet,” and the expected impact and bridge expected impact were calculated by correlation stability (CS), with a value greater than 0.5 indicating good stability (Epskamp et al., 2018).

#### 2.3.3 Web comparison

Based on a previous study (Jin et al., 2022), we assessed the differences in network features across genders. The overall strength of each edge and each strength was evaluated using the Network Test Comparison (NCT) in the R package (Van Borkulo et al., 2017).

## 3 Results

### 3.1 Study sample

A total of 5,130 empty nester were included in this study. There were 2,707 males and 2,423 females with a mean age of 80.01 years (SD = 9.34). The mean CESD-10 score for the entire sample was 7.35 (SD = 4.53) and the mean GAD-7 score was 1.38 (SD = 2.69) (Table 1). Furthermore, 479 patients (10%) scored > 5 on the GAD-7 and were identified as having anxiety symptoms among elderly people living alone, while 1,380 patients (26.9%) score ≥ 10 on the CESD-10 and were identified as having depressive symptoms.

**Table 1:** Sample characteristics (*n* = 5,130).

Characteristics	*n* (%)/mean ± SD
Age (years)	80.01 ± 9.34
Gender	
Men	2,707(52.77%)
Women	2,423(47.23%)
Anxiety symptoms	1.38 ± 2.69
Depressive symptoms	7.35 ± 4.53

### 3.2 Network structure

**Figure 2:**
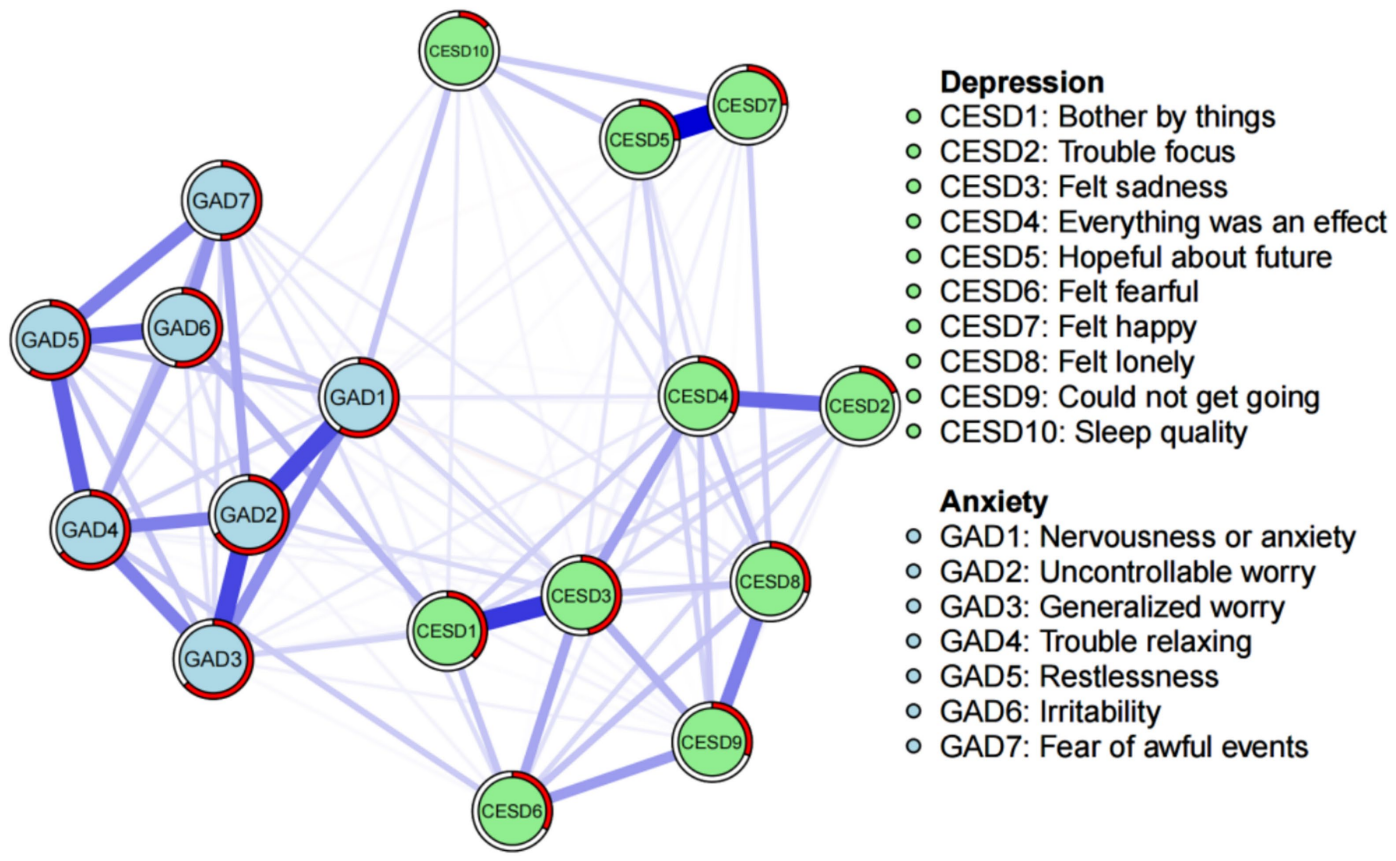
Network structure of depression and anxiety symptoms in empty nester.

The depression and anxiety symptom network for empty nester is shown in Figure 2. The circular pie chart around each node shows the predictability of each symptom. The mean predictability was 0.4156, which indicates that the surrounding nodes typically explained 41.56% of the variance of each node. The correlation matrix for the CESD-10 and GAD-7 items is shown in Supplementary Table S1. The means, standard deviations, and predictability of all CESD-10 and GAD-7 items are shown in Table 2. The most strongly correlated edges among the symptoms of the depression network were CESD-1 (I was bothered by things that do not usually bother me) and CESD-3 (I felt sadness), the CESD-2 (I had trouble keeping my mind on what I was doing) and CESD-4 (I felt everything I did was an effort), CESD-5 (I felt hopeful about the future) and CESD-7 (I was happy), CESD-8 (I felt lonely) and CESD-9 (I could not get “going”). The strongest edges among the symptoms of the anxiety network were GAD-1 (Feeling nervous, anxious, or on edge) and GAD-2 (Not being able to stop or control worrying), GAD-2 (Not being able to stop or control worrying) and GAD-3 (Worrying too much about different things), GAD-4 (Trouble relaxing) and GAD-5 (Being so restless that it is hard to sit still), and GAD-5 (Being so restless that it is hard to sit still) and GAD-6 (Becoming easily annoyed or irritable). The strongest edges in the depression and anxiety network of symptoms were GAD-6 (Becoming easily annoyed or irritable) and CESD-1 (I was bothered by things that do not usually bother me), GAD-3 (Worrying too much about different things) and CESD-1 (I was bothered by things that do not usually bother me), GAD-4 (Trouble relaxing) and CESD-6 (I felt fearful), and GAD-1 (Feeling nervous, anxious, or on edge) and CESD-10 (How is your sleep quality now).

**Table 2:** Descriptive statistics for CESD-10 and GAD-7 items.

Items	Items context	Content abbreviation	Mean	SD	Predictability
CESD1	I was bothered by things that do not usually bother me.	Bother by things	0.34	0.63	0.374
CESD2	I had trouble keeping my mind on what I was doing.	Trouble focus	0.63	0.83	0.188
CESD3	I felt sadness.	Felt sadness	0.28	0.58	0.469
CESD4	I felt everything I did was an effort.	Everything was an effect	0.72	0.85	0.323
CESD5	I felt hopeful about the future.	Hopeful about future	1.45	1.10	0.251
CESD6	I felt fearful.	Felt fearful	0.23	0.53	0.328
CESD7	I was happy.	Felt happy	1.68	1.18	0.248
CESD8	I felt lonely.	Felt lonely	0.38	0.71	0.295
CESD9	I could not get “going.”	Could not get going	0.17	0.48	0.306
CESD10	How is your sleep quality now	Sleep quality	1.47	0.93	0.133
GAD1	Feeling nervous, anxious, or on edge	Nervousness or anxiety	0.30	0.55	0.585
GAD2	Not being able to stop or control worrying	Uncontrollable worry	0.21	0.49	0.666
GAD3	Worrying too much about different things	Generalized worry	0.25	0.53	0.633
GAD4	Trouble relaxing	Trouble relaxing	0.18	0.46	0.643
GAD5	Being so restless that it is hard to sit still	Restlessness	0.15	0.42	0.591
GAD6	Becoming easily annoyed or irritable	Irritability	0.18	0.44	0.529
GAD7	Feeling afraid as if something awful might happen	Fear of awful events	0.12	0.38	0.503

**Figure 3:**
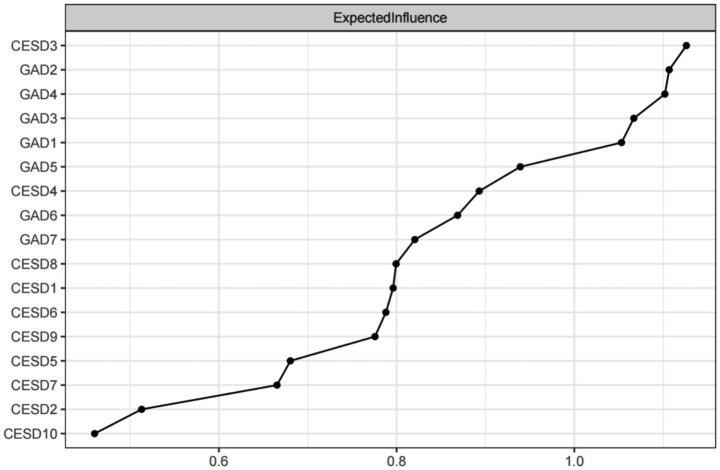
Centrality index: expected impact value.

**Figure 4:**
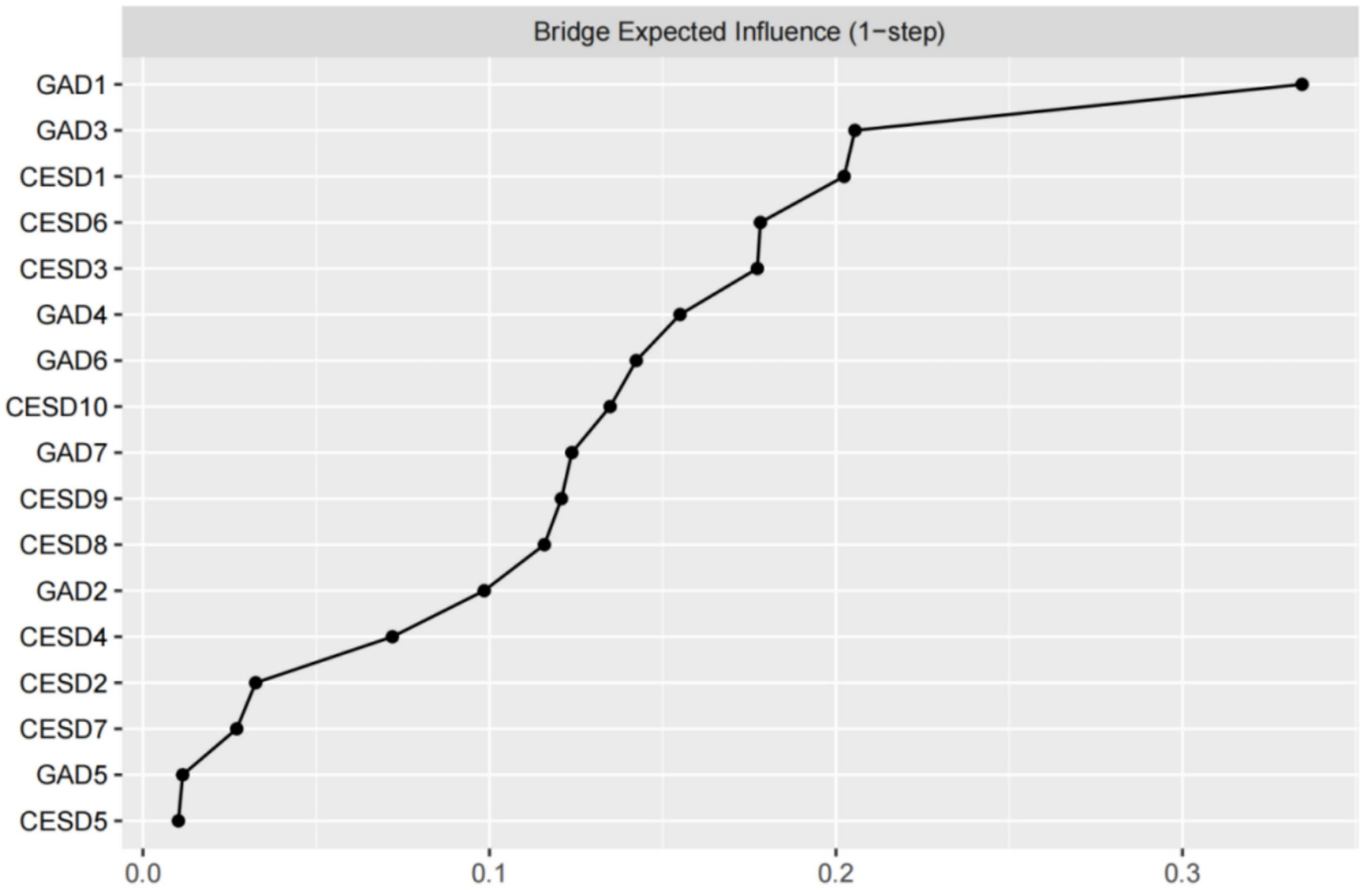
Centrality index: bridge expected influence values.

The EI and bEI values of the symptoms in the depression and anxiety network are shown in Figures 3, 4. The main symptoms were CESD-3 (I felt sadness), GAD-2 (Not being able to stop or control worrying), and GAD-4 (Trouble relaxing) (Figure 3), and it is crucial to understand these three symptoms in order to understand the network of depression and anxiety symptoms in empty nester. The main symptoms connecting the depression and anxiety groups are CESD-1 (I was bothered by things that do not usually bother me), GAD-1 (Feeling nervous, anxious, or on edge), and GAD-3 (Worrying too much about different things) (Figure 4).

### 3.3 Network stability

**Figure 5:**
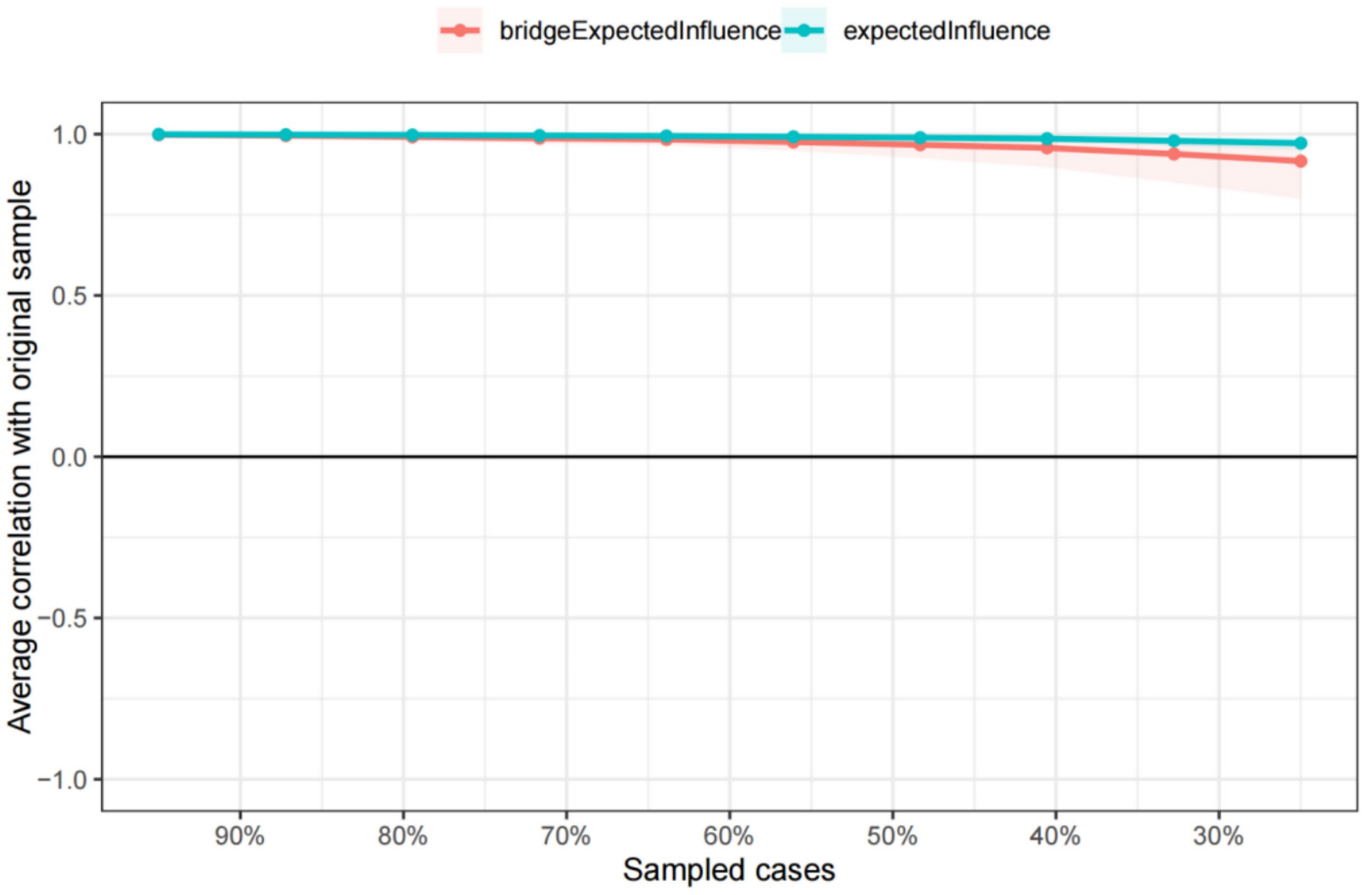
The stability of centrality and bridge centrality indices according to a case-dropping bootstrap method.

**Figure 6:**
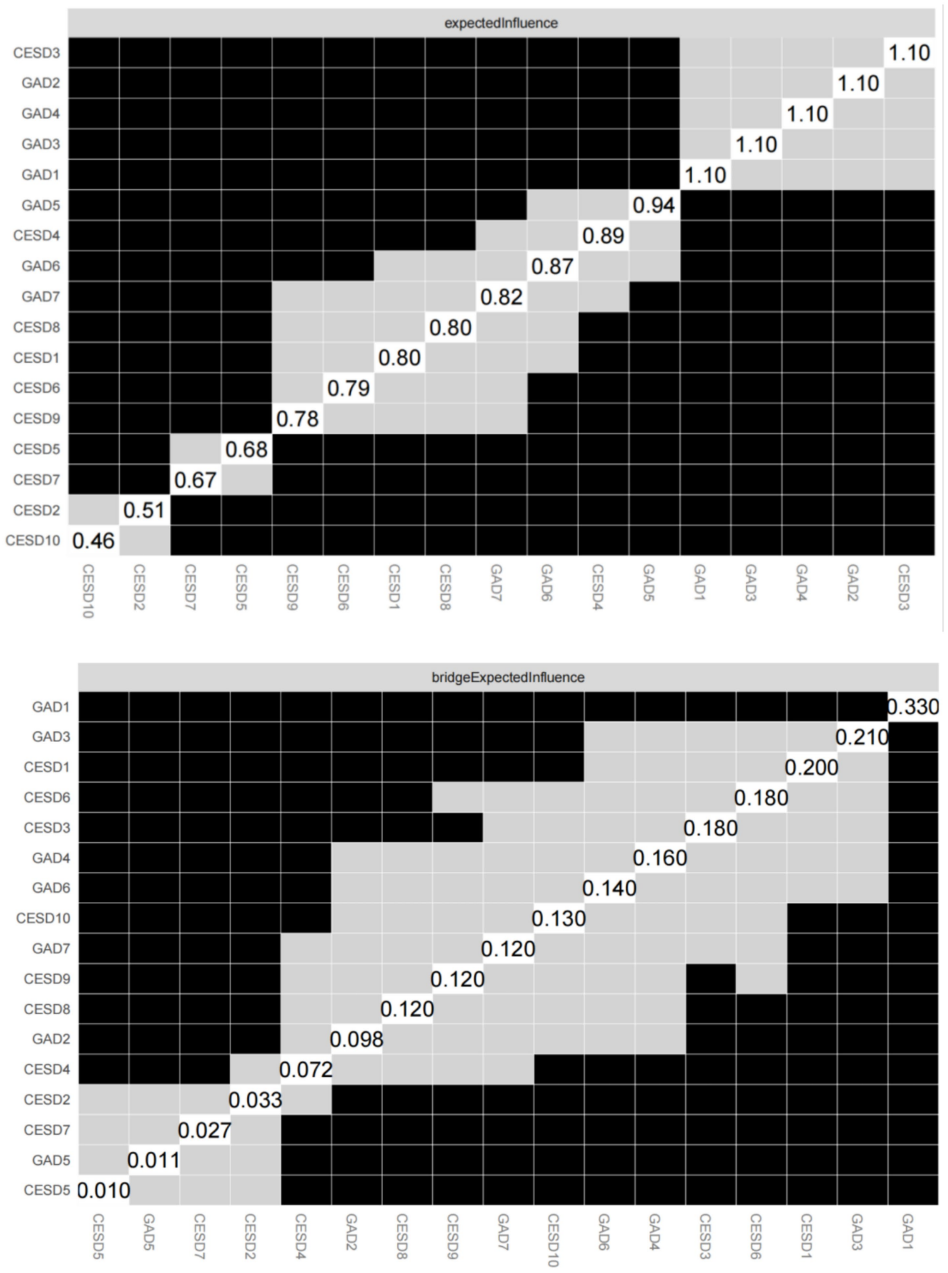
Bootstrapped stability test for “expected influence” and “bridge expected influence”.

The symptoms of the depression and anxiety network have good stability. As can be seen in Figure 5, the stability of the EICS-coefficient is 0.75 and the bEICS-coefficient is 0.75, which indicates that the network does not change significantly when 75% of the samples fall. According to the bootstrap stability test of EI, the central symptom is significantly different from the other nodes (Figure 6). Supplementary Figure S1 shows the results of the non-parametric bootstrap process with a narrow bootstrapped 95% confidence interval, indicating high accuracy.

### 3.4 Comparisons of network structure between the sexes

Supplementary Figures S2, S3 show the network structure for males and females. In a comparison of the network models for female and male empty nester, there were no significant differences in global intensity (males: 7.170, females: 7.221; S = 0.051, *p* = 0.559) or in the distribution of edge weights in the network (M = 0.133, *p* = 0.134) (Supplementary Figures S4, S5).

## 4 Discussion

In this study, the incidence rate of depression symptoms among empty nesters was 26.9%, which is higher than the incidence rate of anxiety symptoms (10%). The same result was also found in Hou B’s study (Hou, 2025). This seems to contradict the widely accepted notion mentioned in the introduction that they have a high comorbidity rate. Several specific factors in our research context may explain this difference. First, the assessment tool itself may have caused this difference; GAD-7 is specifically designed for generalized anxiety disorder and may underestimate other forms of anxiety common among the elderly, such as somatic anxiety or phobias (Spitzer et al., 2006). Second, cultural factors play an important role. In the Chinese cultural context, psychological distress tends to be somatized. Depression symptoms, such as fatigue, sleep disorders, and somatic pain, may be more likely to be reported and considered legitimate health issues, while anxiety may be more internalized, making anxiety symptoms harder to detect.

This study represents the first application of network analysis to examine the relationships between depression and anxiety symptoms in empty nesters, offering novel insights into the complexity of their mental health conditions. Our findings reveal that CESD-3 (I felt sadness)emerged as the most central symptom in the network, followed by GAD-2 (Not being able to stop or control worrying) and GAD-4 (Trouble relaxing). Notably, the key bridge symptoms connecting anxiety and depression were GAD-1 (Feeling nervous, anxious, or on edge), GAD-3 (Worrying too much about different things), and CESD-1 (I was bothered by things that do not usually bother me).

The present study identified CESD-3 (I felt sadness) as the most central symptom in the anxiety-depression network among empty nesters, underscoring its critical role in the interplay between these conditions, making it an effective target for prevention, treatment, and intervention. The Diagnostic and Statistical Manual of Mental Disorders-5 (DSM-5) (2013 edition) (First, 2013) considers sadness as a core symptom of major depressive disorder. When elderly people fall ill or are unable to care for themselves, they are usually taken care of by their children. However, children of empty nester are either away from home or not around for long periods, leaving the elderly without care when they fall ill. Therefore, they not only endure the physical pain of the disease but also the emotional pain of going to the hospital alone, leading to feelings of sadness (Zheng and Zhang, 2024) Notably, prior studies align with these findings: a study of older Chinese adults with hypertension also identified CESD-3 (I felt sadness) as the primary central symptom in a similar network (Ma et al., 2024), while research on older adults with disabilities likewise found it to be the most central symptom in the anxiety-depression network (Zhang P. et al., 2023). Together, these findings reinforce that CESD-3 (I felt sadness) is a pivotal symptom in older populations, highlighting its transdiagnostic significance across different subgroups. Based on the findings of this study, we believe that future care for empty-nest elders should prioritize providing ample companionship and emotional support. Additionally, organizing the elderly to participate in various interest groups (such as chess, singing, etc.) to promote their social engagement is also crucial.

This study identified GAD-2 (Not being able to stop or control worrying) and GAD-4 (Trouble relaxing) as additional central symptoms in the network structure. Notably, these findings contrast with previous research where GAD-1 (Feeling nervous, anxious, or on edge) demonstrated the highest centrality value (Bian et al., 2024), and studies of cataract patients that recognized GAD-2 as central but did not identify GAD-4 as significant (Zhang et al., 2024). These discrepancies likely reflect population-specific variations in symptom networks, highlighting the unique psychopathological profile of Chinese empty nesters. Uncontrollable worry is a cognitive process, typically manifested as repetitive thinking about potential risks. The physical functions of empty nesters gradually decline with age, leading to increased concerns about their own and their spouse’s health (Wang et al., 2025). Furthermore, Daneshvari found that empty nesters often report increased anxiety about retirement planning and exhibit greater concern about death-related thoughts when living alone (Daneshvari et al., 2021). These cumulative stressors manifest clinically as persistent uncontrollable worry and an impaired ability to relax, subsequently exacerbating anxiety and depressive symptoms. From a network theory perspective, central symptoms serve as critical hubs within psychopathological networks. Their centrality is evidenced not only by extensive direct connections with other symptoms but also through their weighted influence across the entire network. Consequently, clinical interventions should prioritize these central symptoms as primary treatment targets. Such focused approaches may produce beneficial cascading effects, potentially leading to broader improvements throughout the symptom network. We recommend adopting intervention measures with Chinese characteristics, such as Tai Chi and Qigong, to help alleviate the ‘Unable to stop or control worries’ and ‘Difficulty relaxing’ of empty nesters, ultimately promoting their physical and mental health (Dong et al., 2025).

Bridge expected influence is a valuable tool for identifying dynamic relationships between symptoms, serving as key therapeutic targets. Precision interventions focusing on bridge symptoms may prove particularly effective in treating comorbid disorders. In the current study, GAD-1 (Feeling nervous, anxious, or on edge) and GAD-3 (Worrying too much about different things) emerged as crucial bridge symptoms in the anxiety-depression network among empty nesters, aligning with previous findings in studies of older adults living alone (Chang et al., 2025) and elderly populations during the COVID-19 pandemic (Ventura-León et al., 2022).

Previous research has suggested that excessive tension or anxiety may activate uncontrollable worry in the short term. Cho indicates that within traditional Chinese family structures, older adults often bear significant responsibilities for family decision-making and grandchild care, while the younger generation is expected to respect and care for their elders. This intergenerational dynamic leads many older adults in China to worry intensely about being unable to continue supporting their families due to declining health or financial difficulties, while also fearing that illness or lower socioeconomic status might make them a burden to their children (Cho et al., 2021). This profound concern, rooted in a sense of familial responsibility, may further intensify their anxiety and worry about future uncertainties.

Network analyses and exploratory factor analyses have consistently identified tension as a critical transdiagnostic symptom associated with both anxiety and depression (Bian et al., 2024). Furthermore, age-related declines in cognitive and physical functioning impair older adults’ environmental judgment, adaptability, and life control capabilities, predisposing them to uncontrollable worry and generalized anxiety (Wang et al., 2022). These factors ultimately contribute to the development of comorbid anxiety and depression. Notably, CESD-1 (I was bothered by things that do not usually bother me) emerged as a central bridge symptom in our study, a finding rarely reported in previous research. This unique result may reflect the specific circumstances of empty nesters in the digital age – without children’s support, they face greater challenges in managing daily affairs, leading to increased distress. This finding highlights the need for greater research attention to the “Bother by things” symptom in empty nester populations and calls for deeper investigation into its mechanistic role as a bridge symptom in anxiety-depression comorbidity.

The current study found no gender-based differences in the anxiety-depression network structure among empty-nester older adults, which contrasts with previous findings (Sileo et al., 2022). This observation aligns with the stress-vulnerability model, positing that comparable stressors elicit similar psychological responses. Specifically, both male and female empty-nesters experience parallel challenges—such as loneliness and altered family dynamics—following their children’s departure (Zubin and Spring, 1977), with these factors exerting comparable psychological effects across genders. Furthermore, environmental and social determinants may supersede gender-specific variations, emerging as predominant influences on mental health during this life stage (Mayerl et al., 2024). Consequently, intervention strategies for anxiety and depression in this population should prioritize addressing shared risk factors (e.g., family-social support systems and health status) rather than focusing on gender disparities.

This study has several limitations that should be acknowledged. First, anxiety and depression among the elderly were assessed using self-reported screening tools (CESD-10 and GAD questionnaires), which may be subject to response bias. Future research should incorporate objective measures, such as biochemical markers, to complement self-reported data and enhance validity. Second, due to the cross-sectional design of this study, causal relationships between variables cannot be established. Longitudinal studies are needed to further explore the temporal dynamics and potential causal pathways underlying these associations. Finally, all data were collected through self-reports, which may introduce biases such as social desirability or recall bias. Future studies could benefit from incorporating multiple data sources (e.g., clinical evaluations, caregiver reports) to improve reliability and accuracy.

In summary, this study is the first anxiety-depression network analysis of empty nester to identify CESD-3 (I felt sadness), GAD-2 (Not being able to stop or control worrying), and GAD-4 (Trouble relaxing) as the central symptoms, and GAD-1 (Feeling nervous, anxious, or on edge), GAD-3 (Worrying too much about different things), and CESD -1 (I was bothered by things that do not usually bother me) are the key bridge symptoms. Intervention strategies should start from the above central and bridge symptoms, consider the causes of sadness, tension, uncontrollable worry, and inability to relax in older adults, and develop multidimensional, targeted intervention strategies. For example, applying cognitive therapy based on positive thinking to reduce anxiety and enhance adaptive emotion regulation and mental health conditions. Considering the high dependency of Chinese elderly on their families, strategies should be formulated starting from the family roles. From a social perspective, strengthening community support networks, establishing community activity centers for the elderly, organizing regular social events, and developing neighborhood mutual aid projects to help the elderly relax both mentally and physically. Furthermore, establishing lifelong learning opportunities, offering courses at community senior universities, providing internet skills training, enhancing the elderly’s ability to solve common problems in life on their own, reducing their unnecessary worries, and increasing their sense of self-worth. In terms of family, promote the use of remote communication technologies, discuss future living arrangements, develop contingency plans, clarify medical and care preferences, and meet the emotional needs of the elderly. In summary, the direction of future interventions should start with the central and bridge symptoms, with the government, community and family working together to reduce the sadness of empty nester, enhance their mental health and improve their quality of life.

## Data Availability

The original contributions presented in the study are included in the article/Supplementary material, further inquiries can be directed to the corresponding author/s.
