# Supplementary material for: A network analysis of anxiety and depression symptoms among empty nesters in China
Source: Front Psychol. 2025 Sep 18;16:1667813. doi: 10.3389/fpsyg.2025.1667813 (PMC12488660; doi:10.3389/fpsyg.2025.1667813)
Supplement: Supplementary file 1 [file Supplementary_file_1.pdf]

Supplementary Materials

Table S1. Correlation matrix of the CESD-10 and GAD-7 items

Figure S1. Bootstrapped stability test for 'expected influence'

FigureS2. Network comparison of anxiety and depressive symptoms between genders-Male

FigureS3. Network comparison of anxiety and depressive symptoms between genders-Female

FigureS4 and FigureS5. Comparison of network properties between females and males.

Data availability

Table S1 Correlation matrix of the CESD-10 and GAD-7 items

	CESD1	CESD2	CESD3	CESD4	CESD5	CESD6	CESD7	CESD8	CESD9	CESD10	GAD1	GAD2	GAD3	GAD4	GAD5	GAD6	GAD7
CESD1	0.000																
CESD2	0.064	0.000															
CESD3	0.311	0.062	0.000														
CESD4	0.075	0.235	0.146	0.000													
CESD5	0.000	0.000	0.041	0.052	0.000												
CESD6	0.092	0.062	0.139	0.047	0.003	0.000											
CESD7	0.010	0.000	0.022	0.025	0.398	0.015	0.000										
CESD8	0.006	0.041	0.092	0.102	0.021	0.096	0.077	0.000									
CESD9	0.000	0.018	0.120	0.085	0.070	0.154	0.005	0.201	0.000								
CESD10	0.036	0.000	0.016	0.053	0.085	0.000	0.086	0.047	0.002	0.000							
GAD1	0.020	0.017	0.063	0.040	0.015	0.018	0.021	0.034	0.014	0.093	0.000						
GAD2	0.001	0.001	0.057	0.000	0.000	0.019	0.000	0.000	0.020	0.000	0.283	0.000					
GAD3	0.068	0.008	0.010	0.032	0.000	0.009	0.006	0.040	0.022	0.012	0.175	0.288	0.000				
GAD4	0.000	0.000	0.015	0.000	0.000	0.081	0.000	0.022	0.006	0.031	0.065	0.198	0.197	0.000			
GAD5	0.000	0.000	0.000	0.000	0.000	0.000	0.000	0.000	0.011	0.000	0.089	0.056	0.093	0.245	0.000		
GAD6	0.114	0.006	0.031	0.000	-0.005	0.000	0.000	-0.016	0.012	0.000	0.080	0.045	0.050	0.137	0.244	0.000	
GAD7	0.000	0.000	0.000	0.000	0.000	0.051	0.000	0.037	0.036	0.000	0.027	0.139	0.057	0.105	0.200	0.169	0.000

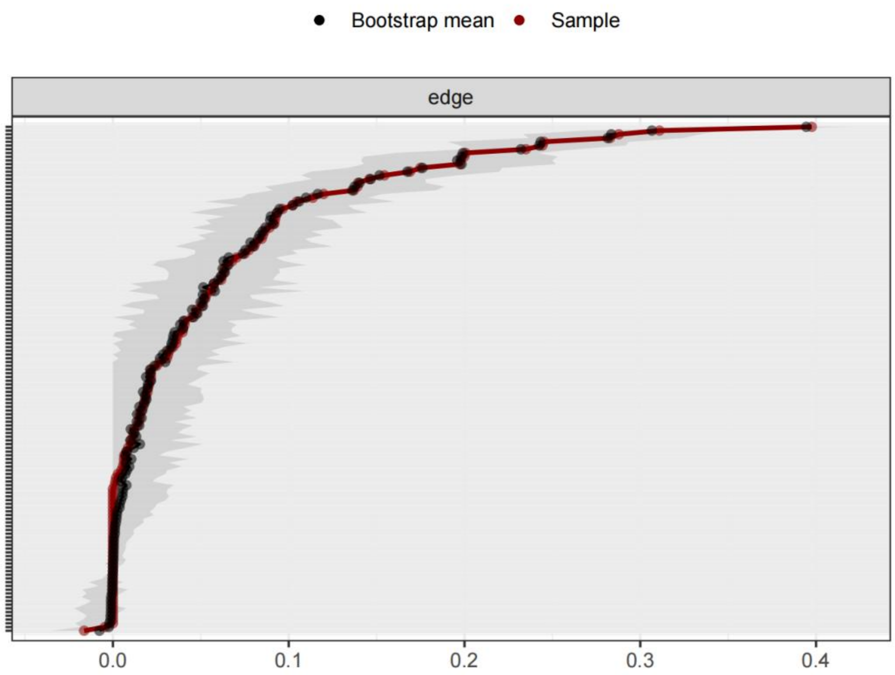

Fig. S1. Bootstrapped stability test for 'expected influence'

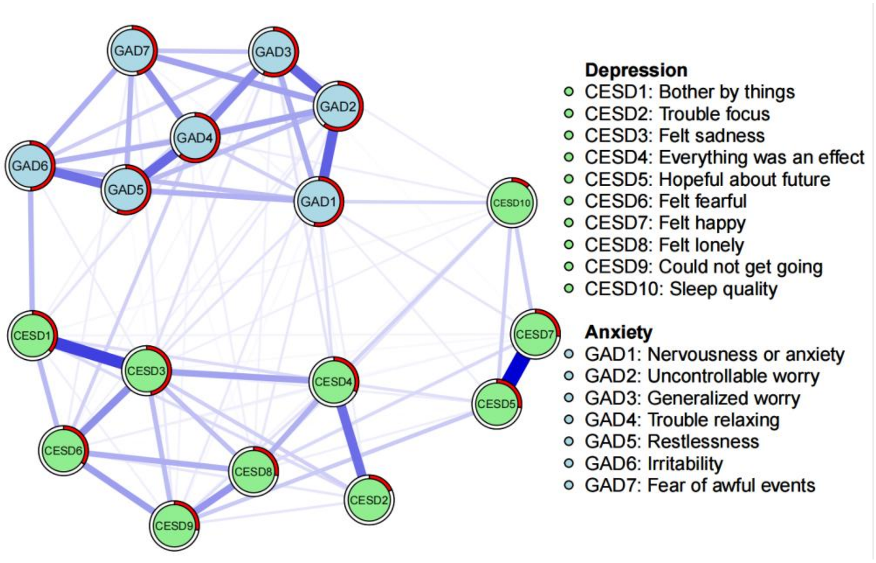

Figure S2: Network comparison of anxiety and depressive symptoms between genders-Male

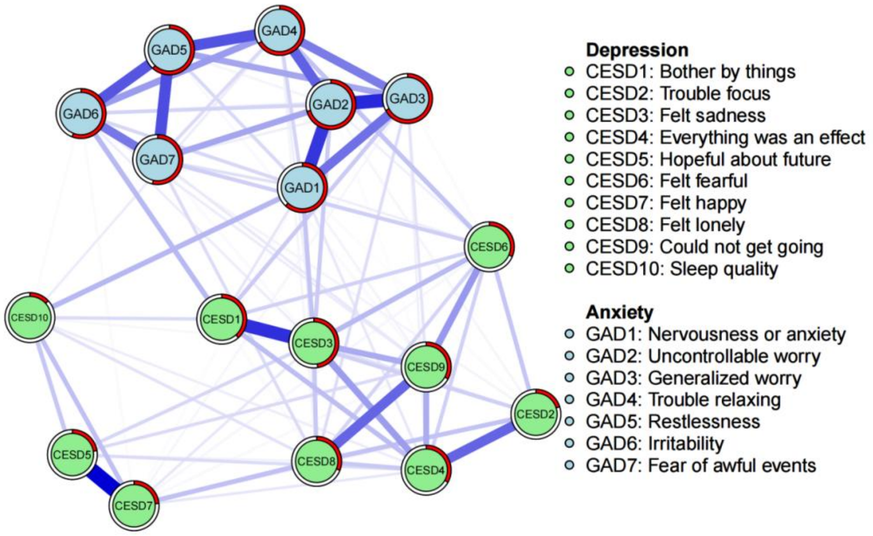

Figure S3: Network comparison of anxiety and depressive symptoms between genders-Female

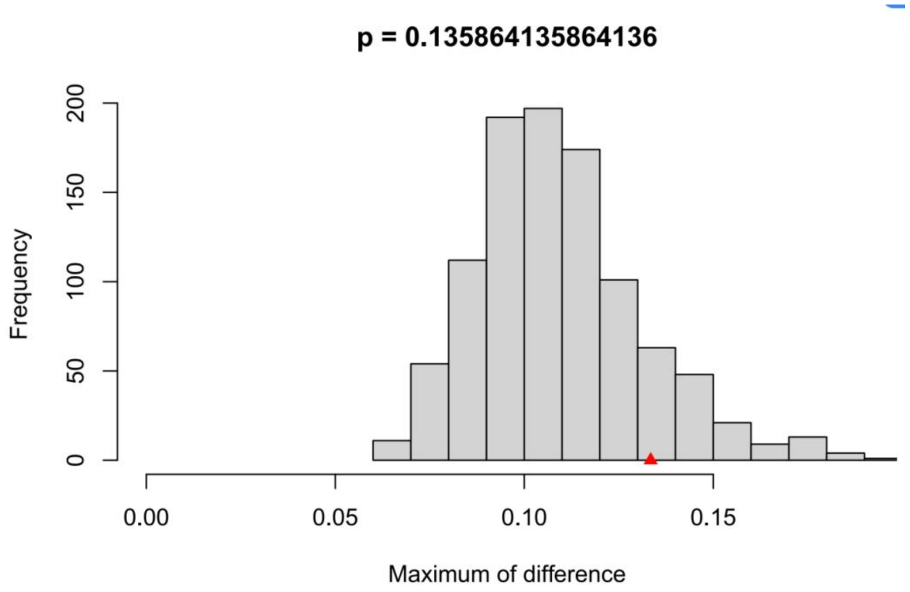

Figure S4 Comparison of network properties between females and males.

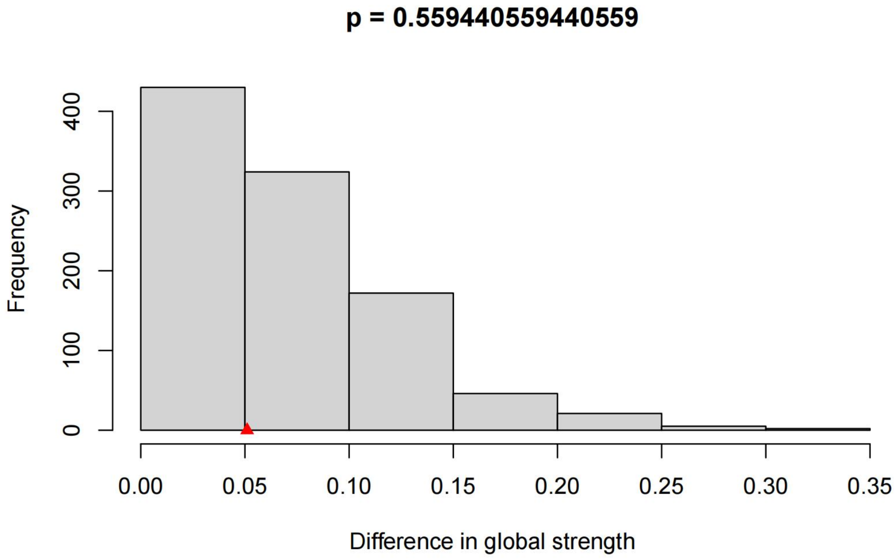

Figure S5 Comparison of network properties between females and males.

Data availability

The analysis in this study utilized publicly available datasets, which are accessible at https://opendata.pku.edu.cn/
